# Mechanisms of Cognitive Change: Training Improves the Quality But Not the Quantity of Visual Working Memory Representations

**DOI:** 10.5334/joc.306

**Published:** 2023-07-17

**Authors:** Shuangke Jiang, Myles Jones, Claudia C. von Bastian

**Affiliations:** 1 Department of Psychology, University of Sheffield, UK

**Keywords:** visual work memory, quantity and quality, training mechanism, capacity and efficiency

## Abstract

As of yet, visual working memory (WM) training has failed to yield consistent cognitive benefits to performance in untrained tasks, despite large improvements in trained tasks. Investigating the mechanisms underlying training effects can help explain these inconsistencies. In this pre-registered, pre-test/post-test online training study, we examined how training affects the quantity and quality of representations in visual WM using continuous-reproduction tasks. *N* = 64 young healthy adults were randomly assigned to an experimental group or an active control group to complete four training sessions of practce in an orientation-reproduction or a visual search task, respectively. We observed that, in the trained task, only the quality, but not the quantity, of visual WM representations significantly increased in the experimental group relative to the control group. These improvements did not generalise to untrained stimuli or paradigms. Therefore, our findings suggest that training gains are not driven by enhanced capacity. Instead, gains in the quality of visual WM representations that are tied to specific stimuli and paradigms may reflect enhanced efficiency in using the existing visual WM capacity.

Working memory (WM) is a cognitive system providing temporary access to representations that are needed for complex cognition in the present moment. WM has a limited capacity of around four chunks of information that can be simultaneously maintained at a time (Cowan, 2001). The individual limit of WM capacity is strongly correlated with reasoning (Conway et al., 2003; Engle et al., 1999; Oberauer et al., 2008), executive functions (Miyake et al., 2000), and a range of other cognitive abilities (for a review, see Barrett et al., 2004). Furthermore, neurocognitive disorders such as ADHD (Martinussen et al., 2005) and age-related cognitive declines (Park et al., 2002) often go along with WM impairments.

The central role ascribed to WM in human cognition has motivated research into training interventions aiming to enhance WM capacity and, thereby, potentially also reasoning and other related cognitive abilities (Jaeggi et al., 2008; Klingberg, 2010; Klingberg et al., 2002). WM training typically involves repeated practice on one or more WM tasks over a short period of time, aiming to improve performance in trained and untrained cognitive tasks. The improvements in related yet untrained cognitive abilities are referred to as transfer effects. However, so far, WM training has failed to yield consistent and robust cognitive benefits (Jaeggi et al., 2012; Karbach & Verhaeghen, 2015; Melby-Lervåg et al., 2016; Morrison & Chein, 2011; Shipstead et al., 2012; von Bastian et al., 2022). Although previous research reported large and replicable gains in the trained WM tasks, transfer effects on untrained tasks remain inconsistent and elusive. A focus on the theoretical mechanisms underlying training gains can yield important insights for when and why transfer effects may occur (Redick, 2019; Smid et al., 2020; von Bastian & Oberauer, 2014).

The capacity-efficiency model of cognitive training and transfer effects (von Bastian et al., 2022; von Bastian & Oberauer, 2014) provides a framework for explaining these inconsistencies in past findings by proposing two, not mutually exclusive, pathways of how training may induce change. One pathway is through expanding cognitive capacity itself. Expanded capacity should generalise to any untrained tasks that draw on the same capacity limit. WM training-induced enhancements of capacity would be reflected by an increased quantity of representations that are simultaneously maintained in WM. These improvements would be expected to yield broad benefits across a range of related cognitive abilities. However, given the lack of broad and robust transfer effects, it is unlikely that training expands working memory capacity (von Bastian et al., 2022).

The other pathway is through enhancing efficiency in using the available capacity. Mechanisms of enhanced efficiency can be broadly grouped into compression and optimisation. Compression is to learn the regularities of information and making use of observed redundancies to reduce the overall cognitive load (Bavelier et al., 2012; Brady et al., 2009). Compression-based efficiency can be *paradigm-specific* through learning the necessary routines and effective strategies for completing an ongoing task. For example, performance can be boosted by strategies such as chunking (e.g., remembering the three digits 8, 1, and 9 as one number 819). In addition, better metacognitive skills, such as improved introspection about self-performance in an ongoing task (Carpenter et al., 2019) could facilitate applying effective task strategies to a different context (Belleville et al., 2014). Compression can also be *stimuli-specific*, for example through gaining a level of perceptual expertise that allows for more efficient coding of the stimuli (Curby & Gauthier, 2007) by increasing the precision of their representations in WM (Scolari et al., 2008). Finally, efficiency can also be enhanced by optimizing attention allocation to different stimuli or task sets (De Simoni & von Bastian, 2018; Zerr et al., 2021). In contrast to the broad benefits that are expected to result from expanding capacity, enhanced efficiency is expected to be useful only in contexts where these efficiency mechanisms can be applied as well.

There is tentative evidence for training-induced enhancements in efficiency. For example, De Simoni and von Bastian (2018) found that the majority of participants reported the acquisition of paradigm-specific strategies during training, including cognitive load-reducing strategies such as remembering only one of two items of a pair in an associative memory task. De Simoni and von Bastian also found that participants improved selectively in remembering which items they have encountered (i.e., item recognition) but not their current context (i.e., item recollection; e.g., the item’s location on the screen). De Simoni and von Bastian speculated that these improvements in recognition were possibly due to training-induced acquisition of stimuli-specific expertise by which the precision of the item representations in memory was enhanced (see also Olson et al., 2005), thereby increasing success of retrieval. In the present study, we focus on investigating to what extent the acquisition of paradigm-specific and stimuli-specific expertise transfers to other contexts. Paradigm-specific expertise may lead to better performance in tasks with the same surface structure but different stimuli (e.g., recall the orientation of triangles or the shape of rings). Stimuli-specific expertise may lead to better performance in tasks using the same stimuli but different paradigms (e.g., the orientation of triangles in a recall or recognition task).

To distinguish training effects through capacity from those through efficiency, WM models that differentiate between the quantity and the quality of representations maintained in WM are useful (Alvarez & Cavanagh, 2004; Awh et al., 2007; Fougnie et al., 2010; Olson & Jiang, 2002; Zhang & Luck, 2008). This distinction between the quantity (the number of remembered items) and quality (the precision of these items) has been supported by neural evidence demonstrating a dissociative role of different parietal-occipital subregions. Specifically, the inferior intraparietal sulcus (IPS) has been found to track the number of items at different locations, whereas the superior IPS and lateral occipital complex encoded the precision of the attended items (Todd & Marois, 2004; Xu & Chun, 2006). Furthermore, WM quantity, but not quality, shows a strong connection with fluid intelligence (Fukuda et al., 2010).

To date, only few existing studies have investigated training-induced changes specifically in the quantity and quality of visual WM representations (Buschkuehl et al., 2017; Moriya, 2019; Ovalle Fresa & Rothen, 2019; Wang & Qian, 2021), and most of the existing studies offer only crude estimates of changes in quantity and quality of visual WM representations. For example, Moriya (2019) distinguished between the quantity and quality of visual WM representations using two versions of change-detection tasks, in which participants were asked to compare two memory arrays and detect whether they are identical or not. Moriya’s tasks varied in the extent to which the deviating stimulus differed from the memoranda: 45° in the quantity version vs. 5° in the quality versions of the task. Moriya found significant effects of training for both the quantity and the quality versions of the change-detection tasks, but with asymmetric patterns of transfer: whereas training of the quantity task led to strong transfer to the quality version, training of the quality task yielded only weak transfer to the quantity version. However, performance changes in quantity and quality of visual WM were estimated by the same parameter (i.e., Pashler’s *k*, 1988) and, thus, conclusion about the two types of visual WM representations could only be drawn indirectly. Similarly, Wang and Qian (2021) reported training effects of the same change-detection paradigm on the quantity of visual WM representations as well as transfer effects on the quality of visual WM representations, measured by a trained orientation-change detection task and an untrained orientation continuous-reproduction task, respectively. However, Wang and Qian measured the quality of visual WM representations using the overall recall error which mixes quantity and quality of visual WM representations.

Buschkuehl et al. (2017) trained participants in one of two variants of a colour-change detection task. Different to the Moriya (2019) and Wang and Qian (2021), Buschkuehl et al. (2017) used transfer tasks that allowed for estimating the precision of WM representations. Despite substantial training improvements in change-detection performance, the authors found no transfer of these improvements to the precision of representations of colour and spatial features. However, like the other existing studies, Buschkuehl et al. did not use training tasks that allowed for distinguishing changes in the quantity from changes in the quality.

Continuous-reproduction tasks, in which participants were asked to memorise and later reproduce features of stimuli on continuous dimensions (e.g., orientation or shape), probe high-resolution contents of visual WM directly (Gorgoraptis et al., 2011; Ma et al., 2014; Wilken & Ma, 2004; Zhang & Luck, 2008). The dependent variable, that is, the difference between the original and the reproduced feature can then be used to estimate the quantity (or capacity) and quality (or precision) of visual WM representations using computational models such as the standard mixture model (SMM; Zhang & Luck, 2008). The SMM assumes a mixture of two components: a uniform distribution representing random guesses, and the standard deviation of a von Mises distribution (a circular normal distribution) around the target, representing that remembered information is remembered with a certain degree of precision. For example, Ovalle Fresa and Rothen (2019) used a continuous colour-reproduction task to train participants in visual long-term memory and applied the SMM. After six training sessions over the course of three days, participants’ precision in both visual long-term memory and visual short-term memory improved significantly. However, Ovalle Fresa and Rothen focused on long-term memory training, and did not assess transfer to substantially different stimuli and paradigms. Therefore, taken together, it remains unclear whether WM training effects are due to changes in quantity or quality of visual WM representations, and to what extent these changes are specific to the trained paradigm or stimuli. The present study fills this gap.

## Present Study

This pre-registered study investigated the mechanisms of training gains by distinguishing between quantity and quality of representations in visual WM. We administered a continuous orientation-reproduction training task for four training sessions. To examine the capacity-efficiency model and its proposed mechanisms of training and transfer effects, we used the SMM (Zhang & Luck, 2008) to estimate changes in the quantity (i.e., capacity) and the quality (i.e., precision) of visual WM representations from pre-test to post-test and during training. Furthermore, we assessed transfer to two untrained tasks (shape reproduction and orientation-change detection). All effects in the experimental training group were evaluated relative to an active control group practising visual search, which has been shown to demand only minimal visual WM (Wolfe & Horowitz, 1998; Woodman et al., 2001). Including an active control group controls for placebo effects and expectancy effects (Foroughi et al., 2016; Simons et al., 2016; von Bastian & Oberauer, 2014).

Our pre-registered hypotheses^1^ (https://osf.io/mk8fa) are summarised in Table 1 and stated as follows:

**Table 1:** Hypotheses.


MECHANISM	TRAINED TASK (ORT)		UNTRAINED STIMULI (SRT)		UNTRAINED PARADIGM (ODT)
		
	QUANTITY	QUALITY	QUANTITY	QUALITY	PERFORMANCE

Capacity	Increase	–	Increase	–	Increase

Efficiency: Paradigm-specific expertise	–	Increase	–	Increase	No change

Efficiency: Stimulus-specific expertise	–	Increase	–	No change	Increase


*Note*: All performance changes are relative to changes observed in the active control group. Hyphens (–) refer to possible concurrent improvements. ORT: orientation-reproduction task; SRT: shape-reproduction task; ODT: orientation-change detection task.

1. If visual WM training-induced performance gains reflect increased visual WM capacity, the experimental group will show larger gains in the quantity of visual WM representations in the trained task (orientation reproduction) and in the untrained, structurally similar task (shape reproduction) as well as improved performance in the untrained structurally different task (orientation-change detection) above and beyond any improvements observed in the active control group.
2. If visual WM training-induced performance gains reflect acquisition of paradigm-specific expertise, the experimental group will show larger gains than the active control group in the quality of visual WM representations in the trained task (orientation reproduction) and in the untrained, structurally similar task (shape reproduction), but no performance gains in the untrained, structurally different task (orientation-change detection).
  If, in addition to these improvements in quality, we would observe training-specific gains in the quantity of visual WM representations in both reproduction tasks, it would suggest that paradigm-specific expertise (e.g., strategies) hindered transfer to the structurally different task. If those training-induced quantity gains were observed in just one of the reproduction tasks, it would suggest that training-induced performance gains were primarily driven by gains in paradigm-specific expertise.
3. If visual WM training-induced performance gains reflect acquisition of stimuli-specific expertise, the experimental group will show larger gains than the active control group in the quality of visual WM representations in the trained task (orientation reproduction) only, without any improvements in the quality of visual WM representations in the untrained, structurally similar task (shape reproduction). If this increased quality of visual WM representations is observed in the trained task but not in the shape-reproduction task, alongside increased visual WM performance in the orientation-change detection task, it would suggest that stimuli-specific expertise transferred across paradigms.
  Importantly, these hypotheses were not mutually exclusive as increases in visual WM capacity and acquisition of stimuli-specific and task-specific expertise may co-occur (von Bastian et al., 2023).

## Method

This online training study used a pre-test-post-test, randomised-controlled design. Participants who had completed the pre-test were randomly assigned to the experimental group or the active control group where they practised an orientation-reproduction task or a visual search task, respectively, for four training sessions. Most participants (87% of the final sample included in the analysis) completed the four training sessions over four consecutive days. Participants who missed a day were retained until they completed their sessions or withdrew. To ensure that participants could maximally complete one training session per day, they received a website link for the next day’s session only after they had completed the previous session. After the training sessions, participants completed the post-test. The pre-test and post-test were designed to assess training effects on performance in the orientation-reproduction task and visual search task, as well as transfer effects to a shape-reproduction task and an orientation-change detection task.

This experiment and its hypotheses were pre-registered on the Open Science Framework (https://osf.io/mk8fa). Pilot data from six participants were collected before the pre-registration. The pilot study served to test the feasibility of the study and the compatibility between the recruitment platform Prolific (https://www.prolific.co) and the experiment software Tatool Web (www.tatool-web.com, von Bastian et al., 2013). As the pilot study was successful with no further changes to the study materials, the pilot data were included in the current study. The study was approved by the University of Sheffield Research Ethics Committee.

### Participants

The target sample size was 100 participants at post-test. An *a priori* power analysis assuming a small to medium within-between interaction effect size (Cohen’s *f* = 0.15) and power of 1-ß = 0.80 suggested a sample size of *N* = 90, which we increased by 10 participants to account for possible dropouts. We recruited 108 healthy participants, aged from 18 to 35, to take part in a study on “Cognitive training” that was advertised on Prolific. We pre-screened participants by customising the allow list according to our pre-registered inclusion and exclusion criteria. After signing up for the study, participants gave online consent to taking part in the study by clicking a button. All participants who met the inclusion criteria and completed the study received £17.40. Before the start of recruitment, a list of group assignments was randomly generated on GraphPad (https://www.graphpad.com/quickcalcs/randomize2/). Following this pre-generated list, participants who completed the pre-test were randomly assigned to either an experimental group or an active control group. Participants were blind to the group condition.

**Figure 1:**
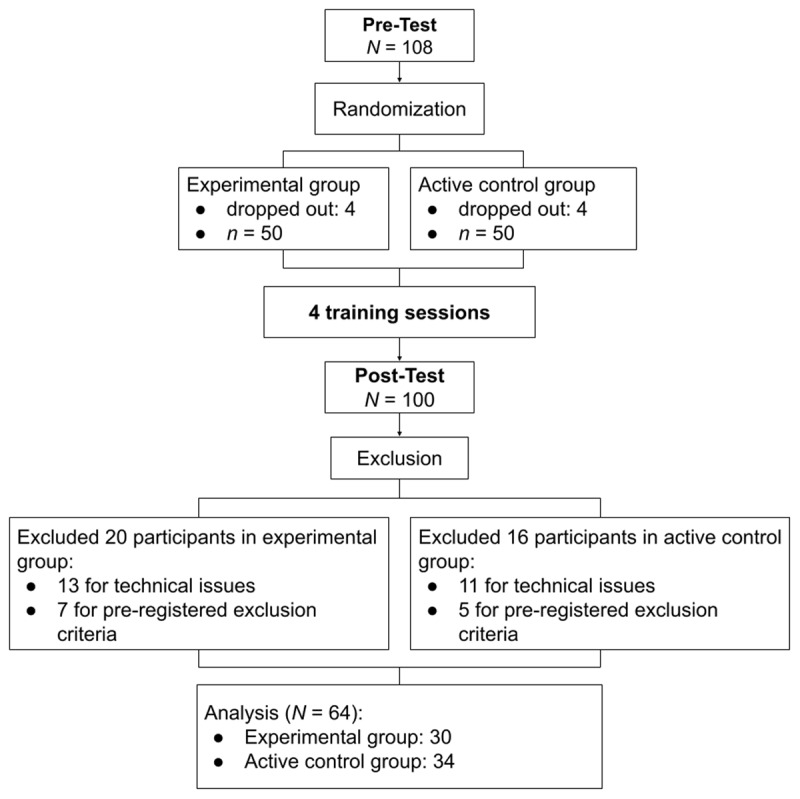
Participant Flow Chart.

The flow chart in Figure 1 illustrates participant recruitment, attrition, and retention. Eight participants (four from each group) dropped out, without giving a specific reason, after completing the pre-test. We replaced these eight participants who dropped out, so that we reached the target sample size of *N* = 100 participants who completed the post-test. After concluding data collection, data from 36 participants were excluded from analysis. Data from two participants in the experimental group were partially missing due to technical issues and, therefore, these data were excluded. In addition, although we instructed them otherwise, we noticed that some participants completed some sessions (pre-test, post-test or training) multiple times. We excluded all participants (11 per training group) for whom the number of additional trials exceeded 10% for any task (12 trials per task). Furthermore, seven participants from the experimental group and five from the active control group were excluded according to pre-registered criteria using reaction times (RT) and omission errors designed to identify participants who did not follow instructions in an online experiment setting.^2^ Of the remaining 64 participants included in the analysis, 30 were in the experimental group and 34 were in the control group. Sensitivity analyses which included all these 12 participants who were excluded due to pre-registered criteria showed similar patterns of results and, thus, led to the same conclusions. Table 2 lists the participants’ demographics. Overall, the groups were comparable regarding their gender and age, but the evidence for the absence of group differences was ambiguous.

**Table 2:** Participant Demographics as a Function of Groups.


MEASURE	GROUP		COMPARISON		
	
	EXPERIMENTAL	ACTIVE CONTROL	STATISTICAL VALUE	*p*	BF_10_ ± ERROR %

Group size: *n*	30	34			

Gender: female/male/non-binary	8/22/0	17/17/0	2.73	.098	3.40 ± 0.00

Age: *M* (*SD*)	22.73 (3.92)	21.94 (2.52)	0.33	.745	1/2.62 ± 0.00


*Note*: Gender differences were tested with a chi-squared test and age differences with Yuen’s t-test.

### Materials

**Figure 2:**
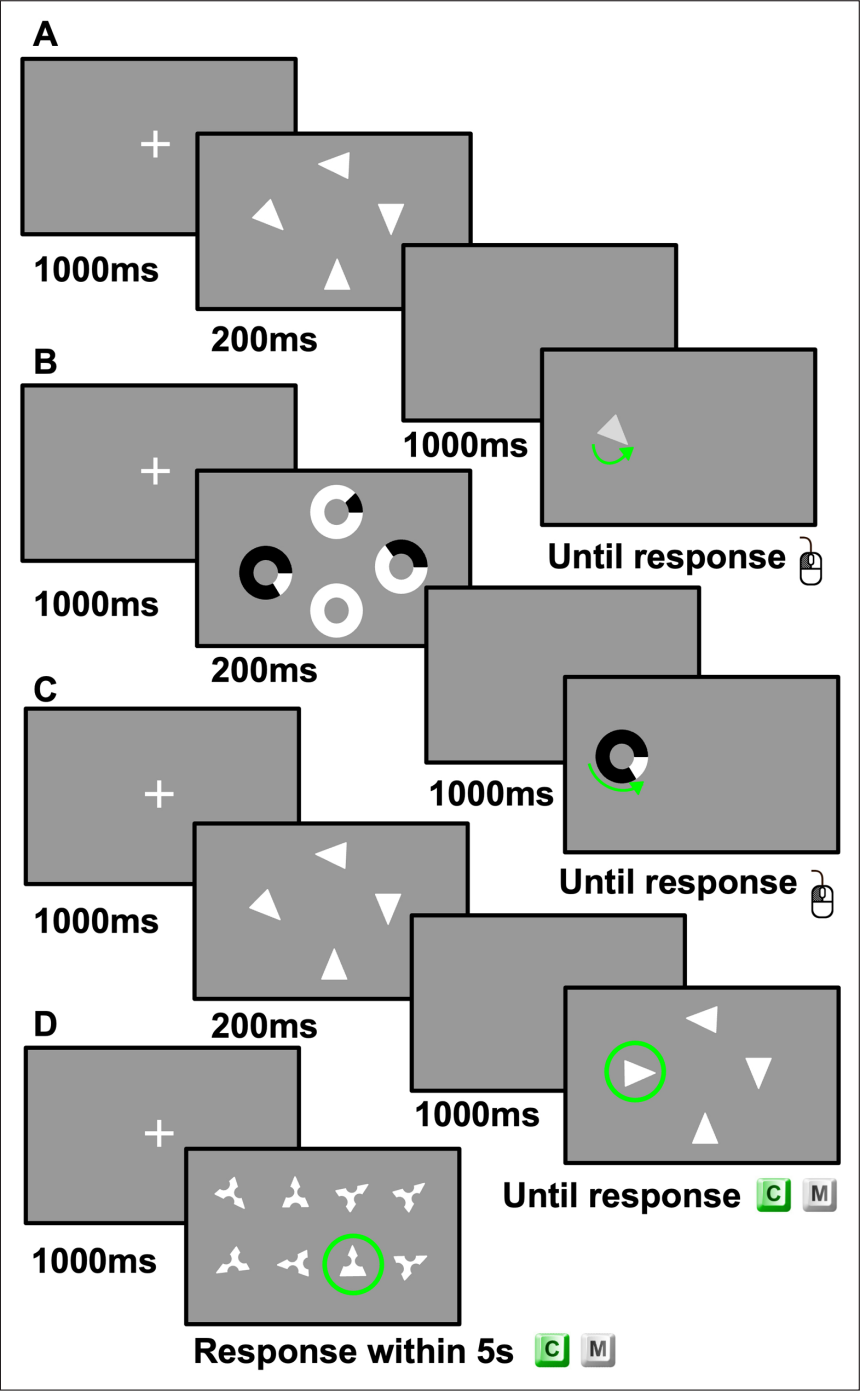
Training and Transfer Tasks. *Note*: Panel A: Orientationreproduction task at set size 4. Panel B: Shape-reproduction task at set size 4. Panel C: Orientation-change detection task at set size 4 in the change condition. Panel D: Visual search task at set size 8 in the change condition.

Figure 2 illustrates the training and transfer tasks. In pre-test and post-test, each experimental task comprised 20 practice trials and 120 testing trials with a set size of the stimulus array of 4 items in the visual WM tasks, and 16 items in the visual search task. The order of representing different experimental tasks was random. Pre-test and post-test took approximately 40 min each. Participants underwent four training sessions. Each training session consisted of 360 trials, with 120 trials per set size (2, 4, and 6 in the orientation-reproduction task, and 8, 16, and 24 in the visual search task). Set sizes were intermixed within each session. Each training session lasted approximately 30 min.

#### Orientation-Reproduction Task

Each trial began with a fixation cross displayed centrally for 1000 ms. Next, an array of randomly orientated (0–360°) isosceles triangles was arranged in a circular manner and appeared on the screen for 200 ms, followed by a 1000 ms blank screen. Then, one of the displayed triangles was randomly selected as the target stimulus and presented in a random orientation. Participants were instructed to reproduce the original orientation by rotating the triangle with the computer mouse and clicking the left mouse-button to record their response.

We measured recall errors, that is, the difference in degrees between the reproduced orientation and the target orientation, ranging from -π to π, to estimate capacity and efficiency parameters by fitting the SMM (Zhang & Luck, 2008) using the MemToolbox (Suchow et al., 2013).^3^ The SMM consists of two components, a von Mise distribution approximating a circular normal distribution, and a uniform distribution:


1 P(x) = \left({1 - g} \right)\frac{1}{{2\pi {I_0}(\kappa)}}{e^{\kappa \cdot cos(x)}} + g\frac{1}{{2\pi }},

where *x* is the response, *g* is the proportion of random guess responses, κ is the concentration parameter of the von Mises distribution, and *I*_0_(κ) is the modified Bessel function of order 0. The SMM assumes that the target can either be recalled with a certain precision or not at all, leading to random guesses. Therefore, the probability of remembering the target (*Pm*) is calculated as


2 Pm = 1 - g.

The quantity of representations in visual WM, that is, capacity *K* is computed as the product of the probability of remembering the target and the set size *N*:


3 K = Pm \times N.

Finally, the quality of representations in WM, that is, precision, is computed as the inverse of the standard deviation (*SD*^–1^) of the von Mises distribution, which was converted from the concentration parameter κ.

#### Shape-Reproduction Task

Following a central fixation cross for 1000 ms, an array of black ring-shaped objects with varying proportions filled in white were distributed on the screen in a circular manner for 200 ms. After a 1000 ms blank screen, one of the displayed objects was randomly selected as the target stimulus. The target stimulus was presented in black colour with a white bar. Participants were instructed to reproduce the original proportion of the white segment by rotating and left clicking the mouse. As for the orientation-reproduction task, capacity and precision were estimated based on the recall errors using the SMM.

#### Orientation-Change Detection Task

After a fixation cross presented centrally for 1000 ms, an array of randomly orientated (0–360°) isosceles triangles appeared on the screen for 200 ms, followed by a 1000 ms blank screen. Immediately afterwards, a second array was presented until response. In half of the trials, the two arrays were identical. In the other half of the trials, one of the triangles in the second array was randomly selected and presented in a randomly selected, different orientation. Participants were instructed to press the ‘C’ or ‘M’ key of the keyboard to respond to a detection of change or match respectively. To measure visual WM capacity, we computed Pashler’s *k* (Pashler, 1988) for whole-display tasks using Equation 1 (Pashler, 1988; Rouder et al., 2011):


4 k = \frac{{H - FA}}{{1 - FA}} \times N,

where *H* and *FA* are the hit and false alarm rates and *N* is the display set size.

#### Visual Search Task

On each trial, participants first saw a fixation cross for 1000 ms. Then, an array of isosceles triangles with two or three semi-circular gaps, pointing to random directions, was presented. In half of the trials, all triangles had three gaps. In the other half of the trials, one of the triangles had only two gaps. Participants were instructed to press the ‘M’ key of the keyboard within 5 s if all triangles had three gaps, or to press the ‘C’ key if one of the triangles only had two gaps. The overall accuracy which is calculated by the proportion of correct responses excluding omission errors (no response given after 5000 ms), as well as the mean reaction time (RT) for correct responses were measured and used for analysis.

## Results

In addition to frequentist significance tests (including t-tests and analyses of variance, ANOVAs), Bayes factors (BFs) using the default priors from the BayesFactor package (Cauchy distribution with *r* = 0.5 for ANOVAs, *r* = 0.707 for t-tests; Poisson distribution for chi-square tests with *a* = 1) were calculated to evaluate the strength of evidence for the absence or presence of effects (Ly et al., 2016; Rouder et al., 2012). Table 3 lists the categorical labels for describing the strength of evidence adapted from Wetzels and Wagenmakers (2012). As most of the data violated the assumption of normality, we ran robust Yuen t-tests (Yuen, 1974) and report Algina-Keselman-Penfield robust effect sizes, δ_t_ (Algina et al., 2005). We calculated and report both general effect sizes, \eta _{\rm{G}}^2 and partial effect sizes, \eta _{\rm{p}}^2, for ANOVAs to facilitate further use in power analyses and meta-analyses (Lakens, 2013). All statistical analyses were performed with R Statistical software (v4.1.3; R Core Team, 2022). The R packages rstatix (Kassambara, 2021) and ez (Lawrence, 2016) were used for frequentist significance tests. BayesFactor (Morey & Rouder, 2021) and WRS2 (Mair & Wilcox, 2020) were used for Bayesian and robust statistical tests.

**Table 3:** Categorical Labels for Describing the Strength of Bayesian Evidence.


BAYES FACTORS		CATEGORICAL LABELS

H_10_	H_01_	

>100	<1/100	Decisive

30 to 100	1/100 to 1/30	Very strong

10 to 30	1/30 to 1/10	Strong

3 to 10	1/10 to 1/3	Substantial

1 to 3	1/3 to 1	Ambiguous

1	1	No evidence


*Note*: Adapted from Wetzels and Wagenmakers (2012). H_10_ = evidence in favour of the alternative hypothesis; H_01_ = evidence in favour of the null hypothesis.

### Training Performance

Table 4 lists the descriptive statistics for the experimental group and the active control group in the orientation reproduction and visual search tasks during training. To analyse performance changes during training, we ran a repeated-measures ANOVA with the within-subjects factors Time (training session 1 to 4) and Set Size (2, 4, 6).

**Table 4:** Descriptive Statistics of Performance During Training.


MEASURE	TRAINING SESSION							

	1		2		3		4	
			
	*M*	*SD*	*M*	*SD*	*M*	*SD*	*M*	*SD*

Experimental Group (*n* = 30)								

Capacity (*K*)								

Set Size 2	1.88	0.15	1.88	0.19	1.89	0.12	1.89	0.16

Set Size 4	2.76	0.71	2.92	0.74	2.93	0.73	2.87	0.79

Set Size 6	2.97	1.29	3.15	1.30	3.28	1.29	3.25	1.33

Precision (*SD*^–1^)								

Set Size 2	0.08	0.02	0.09	0.02	0.09	0.02	0.09	0.02

Set Size 4	0.06	0.02	0.07	0.02	0.07	0.02	0.07	0.02

Set Size 6	0.06	0.01	0.06	0.02	0.07	0.02	0.07	0.02

Active Control Group (*n* = 34)								

Accuracy								

Set Size 8	0.91	0.07	0.92	0.09	0.94	0.05	0.93	0.05

Set Size 16	0.84	0.07	0.84	0.10	0.87	0.08	0.86	0.08

Set Size 24	0.73	0.09	0.73	0.09	0.77	0.09	0.77	0.09

RT (ms)								

Set Size 8	2056	326	1998	305	1890	282	1918	321

Set Size 16	2871	422	2806	401	2712	453	2707	428

Set Size 24	3257	468	3192	452	3101	499	3066	447


*Note*: Capacity ranges from 0 to the set size; precision ranges from 0 to ∞. RT = mean reaction time.

#### Orientation Reproduction

**Figure 3:**
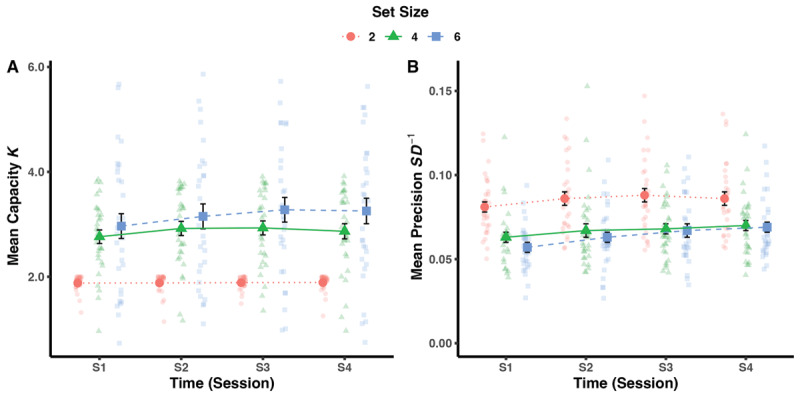
Estimates of Capacity and Precision in the Experimental Group Over Four Training Sessions. Note. Panel A: Estimates of capacity. Panel B: Estimates of precision. Data points with reduced opacity show individual estimates, solid data points represent group means. S1 to S4 = training session 1 to 4.

Figure 3 illustrates estimates of capacity and precision in the experimental group for each training session at set size levels 2, 4, and 6. There was a significant effect of Set Size on both capacity, *F*(2,58) = 39.28, *p* < .001, \eta _{\rm{G}}^2 = .49, \eta _{\rm{p}}^2 = .58, BF_10_ > 100 ± 0.66%, and precision, *F*(2, 58) = 46.12, *p* = < .001, \eta _{\rm{G}}^2 = .37, \eta _{\rm{p}}^2 = .61, BF_10_ >100 ± 0.67%. We observed a significant effect of Time on precision, *F*(3,87) = 6.56, *p* < .001, \eta _{\rm{G}}^2 = .06, \eta _{\rm{p}}^2 = .18, BF_10_ = 3.03 ± 0.59%, but not on capacity, *F*(3,87) = 2.17, *p* = .097, \eta _{\rm{G}}^2 = .01, \eta _{\rm{p}}^2 = .07, BF_10_ = 1/34.51 ± 0.83%. Furthermore, there was no interaction between Time and Set Size for capacity, *F*(6, 174) = 1.94, *p* = .078, \eta _{\rm{G}}^2 = .01, \eta _{\rm{p}}^2 = .06, BF_10_ = 1/60.01 ± 2.56%, or precision, *F*(6, 174) = 0.97, *p* = .444, \eta _{\rm{G}}^2 = .01, \eta _{\rm{p}}^2 = .03, BF_10_ = 1/41.37 ± 1.73%. Taken together, we observed an effect of Set Size on capacity and precision that replicates the set size effect typically observed in visual WM, that is, the bigger the set size, the lower the probability of retrieving an item and its precision. In addition, there was only substantial evidence for significant performance improvement in precision during training.

#### Visual Search

During visual search training, there was a significant effect of Set Size on both accuracy, *F*(2,66) = 154.73, *p* < .001, \eta _{\rm{G}}^2 = .68, \eta _{\rm{p}}^2 = .82, BF_10_ > 100 ± 0.81% and mean RTs, *F*(2,66) = 330.18, *p* < .001, \eta _{\rm{G}}^2 = .82, \eta _{\rm{p}}^2 = .91, BF_10_ > 100 ± 6.87%. We also observed an effect of Time on accuracy, *F*(3,99) = 8.50, *p* < .001, \eta _{\rm{G}}^2 = .08, \eta _{\rm{p}}^2 = .20, with, however, ambiguous Bayesian evidence, BF_10_ = 1/1.36 ± 0.85%, and mean RTs, *F*(3,99) = 7.54, *p* < .001, \eta _{\rm{G}}^2 = .08, \eta _{\rm{p}}^2 = .19, BF_10_ = 1/6.99 ± 0.49%. Furthermore, there was no interaction between Time and Set Size for accuracy, *F*(6,198) = 1.32, *p* = .249, \eta _{\rm{G}}^2 = .01, \eta _{\rm{p}}^2 = .04, BF_10_ = 1/61.71 ± 1.73%, or mean RTs, *F*(6,198) = 0.48, *p* = .823, \eta _{\rm{G}}^2 < .01, \eta _{\rm{p}}^2 = .01, BF_10_ = 1/143.26 ± 2.27%. Taken together, we observed the set size effect in visual search with ambiguous evidence for performance improvements during training.

### Cognitive Performance Changes from Pre-Test to Post-Test

Table 5 lists the descriptive statistics for the training and transfer tasks administered at pre-test and post-test. First, we tested whether the experimental group and the active control group were comparable at baseline based on their pre-test performance using two-tailed *t*-tests (Table 6). Next, we assessed training and transfer effects by running two-way mixed ANOVAs separately for each dependent variable, with the within-subjects factor Time (pre-test, post-test), the between-subjects factor Group (experimental group, active control group), and their interaction. Table 7 provides an overview of the results of these analyses. For testing our hypotheses, we were primarily interested in the Time × Group interaction.

**Table 5:** Descriptive Statistics of Cognitive Performance at Pre-Test and Post-Test.


VARIABLE	GROUP							

	EXPERIMENTAL				ACTIVE CONTROL			
	
	PRE-TEST		POST-TEST		PRE-TEST		POST-TEST	
			
	*M*	*SD*	*M*	*SD*	*M*	*SD*	*M*	*SD*

Training tasks								

Orientation reproduction								

Capacity (*K*)	2.57	0.77	2.89	0.73	2.35	0.91	2.67	0.69

Precision (*SD*^–1^)	0.06	0.01	0.07	0.02	0.06	0.02	0.05	0.01

Visual search								

Accuracy	0.76	0.14	0.81	0.13	0.78	0.09	0.86	0.10

RT (ms)	2973	849	2985	633	3101	475	2636	509

Transfer tasks								

Shape reproduction								

Capacity (*K*)	2.26	0.76	2.10	0.84	2.22	0.68	2.30	0.71

Precision (*SD*^–1^)	0.05	0.02	0.06	0.03	0.04	0.02	0.04	0.03

Orientation-Change detection								
Capacity (Pashler’s *k*)	2.09	1.12	2.37	0.70	2.05	0.82	2.01	0.72


*Note*: Pashler’s *k* can range from 0 to set size. RT = mean reaction time.

**Table 6:** Statistical Group Comparisons at Baseline.


VARIABLE	*df*	*t*	*P*	δ_t_	BF_10_ ± ERROR %

Training tasks					

Orientation reproduction					

Capacity (*K*)	36.72	0.51	.610	–0.13	1/2.42 ± 0.01

Precision (SD^–1^)	37.72	0.71	.484	–0.18	1/3.91 ± 0.01

Visual search					

Accuracy	29.33	0.30	.766	0.08	1/2.93 ± 0.01

RT (ms)	25.34	0.01	.993	0.00	1/3.06 ± 0.01

Transfer tasks					

Shape reproduction					

Capacity (*K*)	33.55	0.38	.707	–0.10	1/3.84 ± 0.01

Precision (SD^–1^)	38.00	1.11	.274	–0.28	1/2.21 ± 0.01

Orientation-Change detection					

Capacity (K)	37.49	0.54	.595	–0.14	1/3.87 ± 0.01


**Table 7:** Analysis of Variance Effects of Training on Cognitive Performance.


VARIABLE/EFFECT	*F*	*P*	\eta _G^2	\eta _p^2	BF_10_ ± ERROR %

Orientation reproduction					

Capacity					

Time	18.12	**<.001**	.04	.23	> 100 ± 2.04

Group	1.53	.221	.02	.02	1/1.58 ± 1.89

Time × Group	0.00	.974	<.01	<.01	1/4.25 ± 3.26

Precision					

Time	3.05	.086	.01	.05	1/2.78 ± 2.21

Group	5.68	**.020**	.07	.08	2.87 ± 1.60

Time × Group	25.63	**<.001**	.07	.29	> 100 ± 4.11

Visual search					

Accuracy					

Time	24.79	**<.001**	.08	.29	> 100 ± 0.84

Group	2.55	.116	.03	.04	1/1.23 ± 2.06

Time × Group	1.55	.218	.01	.02	1/2.03 ± 4.33

Reaction time					

Time	8.22	**.006**	.03	.12	6.61 ± 0.99

Group	0.67	.417	.01	.01	1/2.80 ± 2.08

Time × Group	9.09	**.004**	.04	.13	10.96 ± 2.40

Shape reproduction					

Capacity					

Time	0.15	.704	<.01	<.01	1/5.18 ± 1.28

Group	0.28	.596	<.01	<.01	1/3.31 ± 0.98

Time × Group	1.36	.249	.01	.02	1/2.23 ± 3.69

Precision					

Time	1.12	.293	.01	.02	1/3.47 ± 1.05

Group	4.72	**.034**	.05	.07	1.63 ± 0.80

Time × Group	1.72	.195	.01	.03	1/1.90 ± 2.33

Orientation-Change detection					

Capacity					

Time	1.83	0.181	0.01	0.03	1/2.79 ± 1.00

Group	1.06	0.306	0.01	0.02	1/1.98 ± 0.55

Time × Group	3.12	0.082	0.01	0.05	1/1.05 ± 2.56


*Note*: BF_10_ = Bayes factor in favour of the alternative hypothesis. Degrees of freedom *df_1_* and *df_2_* were 1, 62 respectively.

#### Baseline Comparisons

There were no significant group differences, though the evidence was ambiguous for capacity in the orientation-reproduction task and precision in the shape-reproduction task, with participants in the active control group showing numerically slightly lower capacity in the former task and lower precision in the latter task at pre-test than participants in the experimental group.

#### Training Effects

##### Orientation Reproduction

**Figure 4:**
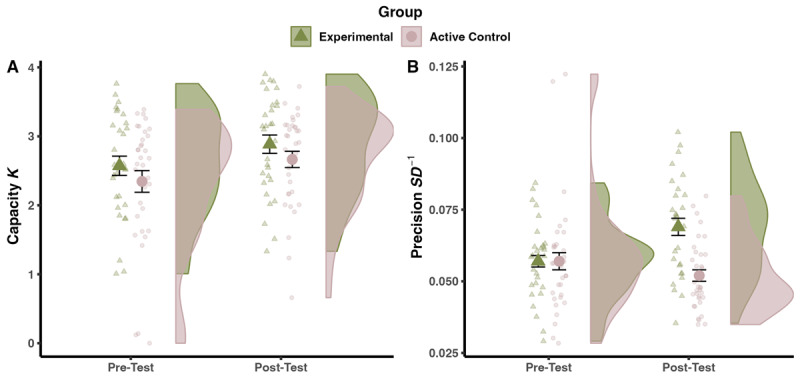
Pre-Post Changes in the Visual WM Training Task on Capacity and Precision. *Note*: Panel A: Changes in capacity. Panel B: Changes in precision. Left: Small transparent data points show the mean values for each individual. Big solid data points show the mean values at group level, with the error bars representing standard errors. Right: Density distributions of the data for both groups.

Figure 4 illustrates the pre-test to post-test changes in capacity and precision in orientation reproduction. The Time × Group interaction was not significant for capacity, *F*(1, 62) < 0.01, *p* = .974, \eta _{\rm{G}}^2 < .01, \eta _{\rm{p}}^2 < .01, with the absence of the interaction being supported by substantial evidence, BF_10_ = 1/4.25 ± 3.26%. These results suggest that training-induced gains cannot be explained by an increase in quantity of representations activated in visual WM.

**Figure 5:**
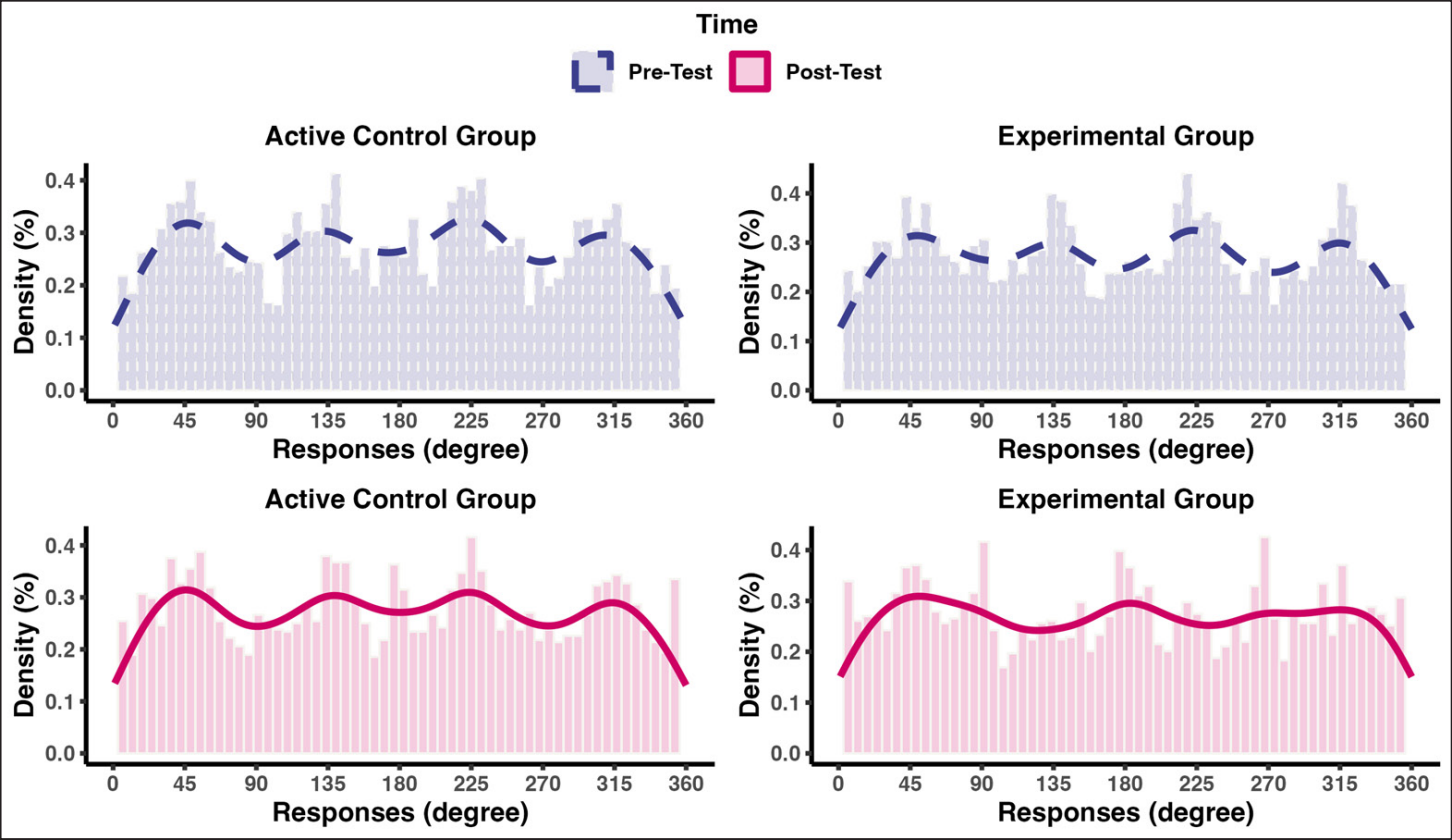
Density of Pre-Post Responses Changes Differs Between Groups. *Note*: Purple histograms with dashed lines show the density of each response at pre-test, and the pink histograms with solid lines show the density of each response at post-test. Number of bins: 60. Experimental group: *n* = 30; active control group: *n* = 34; total responses per participant was 120 each at pre-test and post-test.

For precision, there was a significant Time × Group interaction effect, *F*(1, 62) = 25.63, *p* < .001, \eta _{\rm{G}}^2 = .07, \eta _{\rm{p}}^2 = .29, which was supported by decisive evidence, BF_10_ > 100 ± 4.11%. In the experimental group, precision significantly increased from pre-test (*M* = .06, *SD* = .01) to post-test (*M* = .07, *SD* = .02), *t*(17) = –4.43, *p* < .001, δ_t_ = –1.16, which was supported by decisive evidence, BF_10_ > 100 ± 0.00%. In contrast, in the active control group, precision decreased from pre-test (*M* = .06, *SD* = .02) to post-test (*M* = .05, *SD* = .01), *t*(21) = 1.99, *p* = .059, δ_t_ = .28, though the evidence for this decrease was highly ambiguous, BF_10_ = 1.38 ± 0.02%. Finally, precision was significantly higher in the experimental group than in the active control group at post-test, *t*(28) = 4.36, *p* < .001, δ_t_ = .71, supported by decisive evidence, BF_10_ > 100 ± 0.00%. Taken together, we found considerable training-induced gains in visual WM precision in the trained orientation-reproduction task, with large effect sizes for changes from pre-test to post test and for the comparison to the active control group at the post-test. To further explore the differences in changes between the experimental group and the active control group in the orientation-reproduction task (not pre-registered), we examined the distributions of participants’ responses at pre-test and post-test. As Figure 5 illustrates, we observed a pattern of responses suggesting that, at pre-test, individuals in both groups tended to respond with familiar or canonical orientations, with peaks at 45°, 135°, 225°, and 315°, χ^2^(7, *N* = 7680) = 6.30, *p* = .505, BF_10_ < 1/100 ± 0.00%. At post-test, however, the distribution of responses differed between the groups, χ^2^(7, *N* = 7680) = 44.58, *p* < .001, with decisive Bayesian evidence, BF_10_ > 100 ± 0.00%. Specifically, the experimental group showed a larger number of peaks in their response distribution, leading to a flattened density function and suggesting that, after orientation-reproduction training, participants’ responses included a larger range of finer differences between orientations. In contrast, the active control showed a similar pattern at pre-test and post-test. These observations may indicate that the experimental group was able to distinguish finer differences in orientations after training.

##### Visual Search

For accuracy, the Time × Group interaction was not significant, *F*(1, 62) = 1.55, *p* = .218, \eta _{\rm{G}}^2 < .01, \eta _{\rm{p}}^2 = .02, with the active control group showing a numerically higher accuracy from pre-test to post-test than the experimental group. However, the evidence was ambiguous, BF_10_ = 1/2.03 ± 4.33%. For mean RTs, there was a significant Time × Group interaction effect, *F*(1, 62) = 9.09, *p* = .004, \eta _{\rm{G}}^2 = .04, \eta _{\rm{p}}^2 = .13, which was supported by strong evidence, BF_10_ = 10.95 ± 2.40%. Taken together, participants in the active control group showed larger increases in visual search speed after visual search training than the experimental group, without sacrificing accuracy.

#### Transfer Effects

##### Shape Reproduction

We detected no significant transfer to a task using the same paradigm as the training task but different stimuli. The Time × Group interaction was not significant, *F*(1,62) = 1.36, *p* = .249, \eta _{\rm{G}}^2 = .01, \eta _{\rm{p}}^2 = .02, with, however, capacity decreasing in the experimental group and increasing in the active control group from pre-test to post-test. The evidence for the absence of this interaction was ambiguous, BF_10_ = 1/2.23 ± 3.69%. For precision, the Time × Group interaction was also non-significant, *F*(1,62) = 1.72, *p* = .195, \eta _{\rm{G}}^2 = .01, \eta _{\rm{p}}^2 = .03, with precision, numerically, slightly improving in the experimental group and remaining stable in the active control group. The evidence supporting the absence of the interaction was again ambiguous, BF_10_ = 1/1.90 ± 2.33%.

##### Orientation-Change Detection

Similarly, capacity in a different paradigm but with the same stimuli did not significantly improve after visual WM training. The Time × Group interaction approached significance, *F*(1,62) = 3.12, *p* = .082, \eta _{\rm{G}}^2 = .01, \eta _{\rm{p}}^2 = .05. Numerically, the experimental group performed better at post-test than pre-test, whereas the active control group’s performance remained stable. Again, the evidence for the absence of a transfer effect was near-perfectly ambiguous, BF_10_ = 1/1.05 ± 2.56%. Taken together, there was no transfer to a different type of stimuli or paradigm, with the caveat that the evidence was overall ambiguous.

### Summary

We found evidence for improvements in the trained tasks, with the experimental group improving only in precision, but not in capacity, in the trained orientation-reproduction task, and the active control group improving in RTs in the trained visual search task. Therefore, we rejected Hypothesis 1 that training gains reflect increases in capacity, and we concluded that training gains are driven by increased efficiency. As the improvement in precision did not generalise to performance gains in the untrained shape-reproduction task, we rejected Hypothesis 2 that training gains reflect the acquisition of paradigm-specific expertise, but with the caution that the evidence for the absence of an effect on precision in shape reproduction was ambiguous only. Similarly, there was also no significant effect of orientation-reproduction training on performance in the orientation-change detection task. Therefore, we also rejected Hypothesis 3 that stimulus-specific expertise would transfer to a different paradigm but, again, with the caveat that the Time × Group interaction approached significance, with only ambiguous evidence for the absence of an effect. Therefore, taken together, we found that training gains were stimuli-specific and task-specific, with some ambiguity regarding the potential of these gains in efficiency to generalise to other contexts.

## Discussion

The objective of the study was to identify the mechanisms underlying visual WM training and transfer effects. Specifically, we tested (1) whether training-induced gains after orientation-reproduction training reflect expanded visual WM capacity or enhanced efficiency in using the available capacity by facilitating the acquisition of paradigm-specific or stimulus-specific expertise, and (2) whether such training benefits generalise to other types of stimuli and paradigms. For this purpose, we distinguished training gains in quantity from training gains in quality of visual WM representations and tested transfer effects to an untrained stimulus type (shape reproduction) and paradigm (orientation-change detection).

The results showed that four visual WM training sessions improved the quality of visual WM representations in the trained task but not the quantity. Furthermore, we observed no transfer to different stimuli or a different paradigm. The evidence was ambiguous though, and there was a tendency that the experimental group numerically improved in the orientation-change detection task that used the same stimuli in a different paradigm. Notably, however, if anything, capacity decreased in the experimental group in the shape-reproduction task that uses different stimuli in the same paradigm. Taken together, these findings speak against broad transfer through expanded capacity, which is consistent with the results from other recent WM training studies which reported limited evidence for transfer (Buschkuehl et al., 2017; De Simoni & von Bastian, 2018; Guye & von Bastian, 2017; Redick et al., 2013).

Instead, these findings suggest that training gains are driven by a more efficient use of the available cognitive capacity (von Bastian & Oberauer, 2014; von Bastian et al., 2022). Furthermore, the lack of transfer effects supports the conclusion that the training-induced efficiency gains were both stimuli-specific and paradigm-specific: neither stimuli-specific expertise nor paradigm-specific expertise were generalisable to the same paradigm with different stimuli or a different paradigm with the same stimuli. More specifically, the untrained shape-reproduction task used the same paradigm as the trained visual WM task but tested the memory of shapes instead of orientations. The lack of transfer to this task suggests that training gains reflect gains in expertise in orientation discrimination which is specific to the stimuli employed in the trained task. Yet, the untrained orientation-change detection task used the same stimuli as the trained visual WM task and also tested memory of orientations, but we still did not observe any transfer. However, different to the trained paradigm, the untrained orientation-change detection task might capitalise on configural information, such as the internal representation of the relationship between all displayed orientations at the maintenance stage (Boduroglu et al., 2009; Buschkuehl et al., 2017). At the same time, at the recall stage, the task requirement to detect only one changed orientation out of all stimuli displayed could possibly reduce the need to focus on the feature precision of each stimulus. This could explain why efficiency gains in the trained task did not generalise to another visual WM paradigm using the same stimuli type.

An alternative, not necessarily mutually exclusive, possibility is that the training gains in the orientation-reproduction task reflect a more refined motor control in reproducing the triangles’ orientation. However, the trained orientation-reproduction WM task and the untrained shape-reproduction task arguably require a similar degree of refined motor control to reproduce the orientation or shape information, respectively, by rotating and clicking the mouse. Hence, if the observed training gains merely reflected better motor control, we should also have observed improvements in the untrained shape-reproduction task which requires similar levels of fine motor control. The observed lack of such improvements renders this possibility unlikely.

The findings of the present study also provide some indications how stimuli-specific and paradigm-specific expertise may operate and interact. Our exploratory inspection of response distributions showed that the experimental group but not the active control group reported a larger number of different orientations after training, suggesting that training in the orientation-reproduction task may have catalysed the development of perceptual expertise allowing for discriminating finer differences in orientations. This is in line with other research showing that visual WM training can boost perceptual processing (Truong et al., 2022). Improved perceptual processing due to stimuli-specific expertise may enhance the perceived perceptual distinctiveness (Olson et al., 2005). Given the premise that the active control group’s visual search training involved only little memory (Wolfe & Horowitz, 1998) while sharing similar encoding processing (Kong & Fougnie, 2019), the fact that we observed these precision gains only in the experimental group supports the conclusion that visual WM training-induced gains in efficiency operate at maintenance and recall stage. These stimulus-specific efficiency gains allow for maintaining more precise internal feature representations, and/or discriminating these representations with higher resolution when recalling this feature information.

Developing stimuli-specific, perceptual expertise may also help to use effective paradigm-specific strategies that operate at maintenance and recall stage. Specifically, we found that the experimental group did not only respond a larger number of orientations but more peaks with canonical orientations after training. Participants may have used canonical orientations as a memory aid for the orientations (e.g., 90, 180, and 270 degrees like the numbers 3, 6 and 9 on a clock face). Increasing the number of available canonical orientations may benefit the effectiveness of such a strategy and increase overall performance. Note that this does not exclude the possibility that both experimental and active control training could have improved sensory discrimination at encoding stage.

### Limitations

One major limitation of the current design is that the orientation-change detection task – the untrained paradigm using the same stimuli – did not allow for assessing precision (i.e., the quality of visual WM representations). Consequently, our results cannot fully rule out transfer of gains in the quality of visual WM representations to a different paradigm. Future research with a more fine-grained assessment of the stimulus features is required to identify the mechanisms underlying the transferable gains in quality of visual WM representations.

Another potential limitation of this study is that four training sessions might not be intensive enough to induce transferable training gains in the quality of visual WM representations. Indeed, this possibility is consistent with our results that training gains in the quality of visual WM representations were not detected during training but only at post-test. Furthermore, the spacing of the training sessions may not have optimally supported learning. For example, a design with only one session a week may have allowed for better consolidation of learning effects (e.g., see Lampit et al., 2020). Future research is needed to better understand the optimal intensity and spacing of visual WM training interventions.

Moreover, our training tasks were not adaptive, that is, all participants practised all set sizes irrespective of their individual performance. We chose this design to ensure sufficient measurement of all three set sizes for applying the SMM. However, it might have led to a decrease in motivation. A previous study showed no differences between adaptive and non-adaptive training both for motivation and training and transfer gains (von Bastian & Eschen, 2016); however, in that study participants still received performance-based feedback. Such feedback likely encourages better engagement with the daily training sessions and reduces attrition, which could be useful especially in an online setting like the current study.

Finally, we did not assess participants’ training experience, subjective training gains, or strategies they employed, because we aimed at minimizing the administration time for the benefit of participant retention. However, these data could have added important insights regarding the possible mechanisms underpinning the observed training gains (e.g., see De Simoni & von Bastian, 2018; Guye & von Bastian, 2017). Future research would benefit from including self-report measures for advancing understanding of training-induced change in cognitive performance.

## Conclusion

To the best of our knowledge, the findings of the present study are the first to provide evidence from a continuous reproduction task that visual WM training induces stimuli-specific and paradigm-specific gains in the quality but not in the quantity of visual WM representations. These findings support the notion that training enhances cognitive efficiency through the acquisition of expertise but not capacity. A better understanding of how training facilitates a more efficient use of the available visual WM capacity, and how the underlying training benefits are influenced by the characteristics of stimuli and paradigms, will be critical for harnessing the potential benefits of these training benefits.

## Data Accessibility Statement

Data reported in this manuscript were presented at the Virtual Working Memory Symposium, 2021, the 62^nd^ Annual Meeting of the Psychonomic Society, Boston, MA, USA, 2021, the 63^rd^ Annual Meeting of the Psychonomic Society, Boston, MA, USA, 2022, and the Meeting of the Experimental Psychology Society, London, UK, 2023. Materials, data, and analysis scripts are available at https://osf.io/k5hge/.

## Footnotes

Hypotheses 2 and 3 were slightly reworded (while keeping the identical meaning) to facilitate understanding. Furthermore, paradigm-specific expertise was labelled task-specific expertise in the pre-registration.

Participants were excluded with any of the following three data patterns: RT of less than 1500 ms in 1/3 of the trials in the orientation-reproduction task and in the shape-reproduction task; RT of less than 300 ms in 1/3 of the trials in the orientation-change detection task and in the visual search task; omission errors in 1/3 of the trials in the visual search task.

As noted in the pre-registration, we also explored fitting other existing visual WM models, such as, swap model (Bays, 2016), signal discrimination model (Oberauer, 2021), and target confusability competition model (Schurgin et al., 2020), to the data and conducted a series of systematic model comparisons. Overall, the SMM turned out to be the best fitting model for pre-test to post-test changes and, therefore, is reported here. The model comparisons will be reported elsewhere as this would exceed the scope of the present study.
